# EEG-based analysis for pilots’ at-risk cognitive competency identification using RF-CNN algorithm

**DOI:** 10.3389/fnins.2023.1172103

**Published:** 2023-04-21

**Authors:** Shaoqi Jiang, Weijiong Chen, Zhenzhen Ren, He Zhu

**Affiliations:** ^1^College of Information Engineering, Jinhua Polytechnic, Jinhua, Zhejiang, China; ^2^College of Environment and Engineering, Shanghai Maritime University, Shanghai, China; ^3^College of Merchant Marine, Shanghai Maritime University, Shanghai, China

**Keywords:** ship pilotage, cognitive competency, situation awareness, correlation evaluation, feature identification

## Abstract

Cognitive competency is an essential complement to the existing ship pilot screening system that should be focused on. Situation awareness (SA), as the cognitive foundation of unsafe behaviors, is susceptible to influencing piloting performance. To address this issue, this paper develops an identification model based on random forest- convolutional neural network (RF-CNN) method for detecting at-risk cognitive competency (i.e., low SA level) using wearable EEG signal acquisition technology. In the poor visibility scene, the pilots’ SA levels were correlated with EEG frequency metrics in frontal (F) and central (C) regions, including α/β (*p* = 0.071 < 0.1 in F and *p* = 0.042 < 0.05 in C), θ/(α + θ) (*p* = 0.048 < 0.05 in F and *p* = 0.026 < 0.05 in C) and (α + θ)/β (*p* = 0.046 < 0.05 in F and *p* = 0.012 < 0.05 in C), and then a total of 12 correlation features were obtained based on a 5 s sliding time window. Using the RF algorithm developed by principal component analysis (PCA) for further feature combination, these salient combinations are used as input sets to obtain the CNN algorithm with optimal parameters for identification. The comparative results of the proposed RF-CNN (accuracy is 84.8%) against individual RF (accuracy is 78.1%) and CNN (accuracy is 81.6%) methods demonstrate that the RF-CNN with feature optimization provides the best identification of at-risk cognitive competency (accuracy increases 6.7%). Overall, the results of this paper provide key technical support for the development of an adaptive evaluation system of pilots’ cognitive competency based on intelligent technology, and lay the foundation and framework for monitoring the cognitive process and competency of ship piloting operation in China.

## 1. Introduction

Improvements to ship pilots’ situation awareness (SA) in maritime navigation are critical to reducing human errors, which have caused 75–96% of marine accidents over the last few years (Hetherington et al., 2006; Mohammadfam et al., 2019). In recent years, growth in traffic densities, ship speeds, and ship sizes have led to the need to improve pilots’ operational safety (Weng and Yang, 2015). Due to the complexity of marine systems and the increased use of automation and intelligence, it is becoming more difficult for pilots to fully percept and understand the current environmental state and predict changes in near future (Wild, 2011; Stirling et al., 2019). To prevent pilots’ unsafe behaviors in a more effective and practical manner, it is indispensable to identify at-risk cognitive competency combined with emergency situations in pilotage (Mosier et al., 2013). However, the measurement gap is increased by the requirement of pilots, as the task executors, to maintain high SA levels, as well as the individual differences and experiential properties of pilotage (Darbra et al., 2007). Therefore, identifying at-risk cognitive competency (i.e., low SA levels) as a means of preventing human errors is complicated, and is worthy of further investigation in operational situations.

At present, the collision of poor visibility situations continues to account for a major proportion of all accidents resulting in casualties, and studies are focused on determining their relevance through statistical methods (Antao and Soares, 2019). Chauvin et al. (2013) verified that collision accidents were significantly affected by visibility, which is a variable in 56.51% of environmental factors in the human factors analysis and classification system (HFACS). Bye and Aalberg (2018) supported this finding by calculating the ability of different visibility conditions to explain accidents, and concluded that the most significant effect occurs when the visibility drops to less than 0.25 nautical miles. Existing research has effectively evaluated whether poor visibility conditions are more likely to cause accidents than other environmental factors (e.g., water depth, flow rate, etc.), but has not assessed changes in the cognitive state of the pilots in preparation for potential hazards in poor visibility (Endsley, 2015). In this study, the poor visibility was selected as a typical situation, in which the identification of pilots’ low SA levels is necessary. According to the literature, the SA three-level hierarchical structure is one of the most popular conceptual cognitive frameworks, which frames SA into three stages: perception, comprehension, and prediction (Lo et al., 2016). Therefore, the first problem to solve is how to accurately identify pilots’ low SA level with the three-level cognitive frameworks, taking poor visibility scenes as an example (Zhang et al., 2020).

According to the theoretical interpretation of the SA three-level model, eye movements and EEG parameters among physiological indicators are the most effective reflection of an individual’s intrinsic state from the cognitive processes of perception, understanding and prediction, providing the possibility to essentially prevent unsafe behaviors by detecting SA levels dynamically (Zhang et al., 2020; Pei et al., 2022). The correlation between pilots’ eye-tracking metrics and SA levels has been validated in a previous study (Jiang et al., 2021). However, eye-tracking metrics are only acquired through tangible visual behaviors, and there are no consistent conclusions on whether such visual behaviors truly meet the perceptual requirements in the SA model, or whether the information gazed at is effectively understood by the pilot and the actual behavior is taken (di Flumeri et al., 2018). Therefore, it is necessary to conduct research based on the application of EEG acquisition technology to further assist in quantifying the cognitive processes of unsafe behaviors, and the use of pilots’ EEG features for SA identification is the first problem that needs to be addressed.

To ensure consistency in the study of EEG signals in cross-domain applications, the brain is usually divided into different electrode sites based on five regions [frontal (F), central (C), parietal (P), temporal (T), and occipital (O)] (Aggarwal and Chugh, 2022). EEG is an active map of the brain displayed by converting EEG data to the time-frequency by appropriate methods such as Fourier transform (FT) (Srinivasan et al., 2019). In time-frequency analysis, information about the cognitive state of an individual is usually obtained based on the data in different frequency bands, commonly including delta (δ) (1–4 Hz), theta (θ) (5–8 Hz), alpha (α) (9–14 Hz), beta (β) (15–30 Hz), and gamma (γ) (31–59 Hz) (Pan et al., 2021). Therefore, it has become a common trend to analyze frequency features for the performance evaluation in EEG-based studies (Gutiérrez and Ramírez-Moreno, 2016). Puma et al. (2018) explored the effect of task difficulty on the θ and α bands by varying the number of sub-tasks and showed that the higher the level of the operator’s behavioral performance, the lower the power spectral density (PSD) of the θ and α bands. Dimitriadis et al. (2010) found that the δ band was correlated with the difficulty of the operator’s perceptual task, i.e., the PSD of the δ band increased when the operator perceived the experimental task as too easy. However, previous studies not fully utilizing EEG time-frequency features for cognitive studies.

No common agreement has been made for the EEG features correlated with SA as EEG acquisition techniques were applied in different tasks and workplaces. Regarding the relationship between time-frequency features and SA, there have been attempts to validate the association (Klaproth et al., 2020; Iqbal et al., 2021). Kästle et al. (2021) identified the correlations between β and γ in parietal and temporal regions and SA levels during a control task. Notably, only a few studies (Saini et al., 2020) have adopted multiple EEG spatial-frequency features, but there is no consensus on which features are associated with SA due to different task conditions and application purposes, let alone the identification of pilots’ SA levels based on EEG association metrics.

Therefore, the aims in this paper are twofold: First, the primary goal is to assess the relationships between EEG time-frequency features and SA levels. Correlation analysis indicated that α/β, θ/(α + θ), and (α + θ)/β frequency bands are significantly correlated with SA levels. The ultimate intention of this work is to explore the SA identification method based on associated EEG metrics, which is expected to reduce piloting risks through improved pilot selection and training.

## 2. Experiments and methods

**Figure 1:**
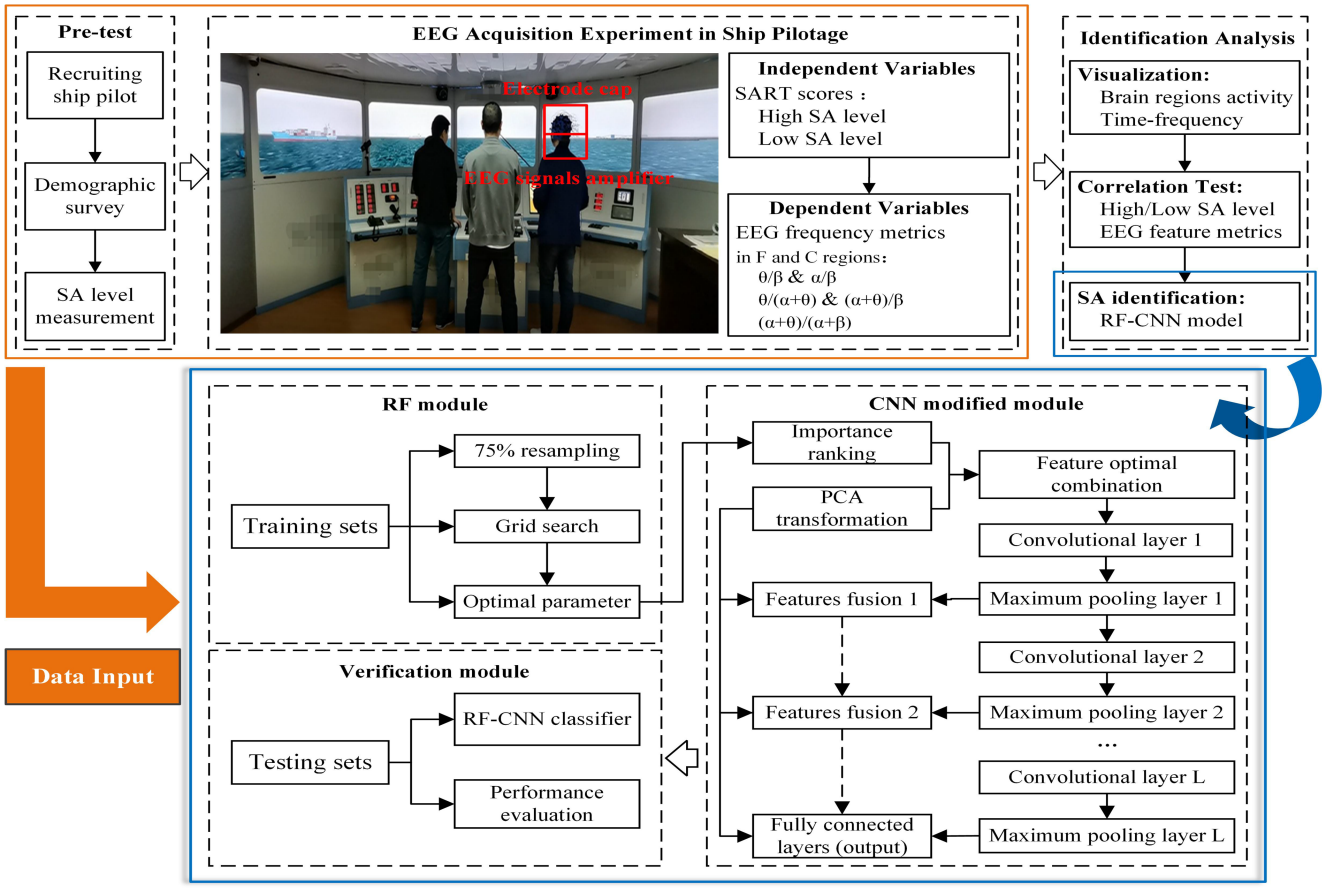
Identification framework of SA levels in the maritime pilotage simulations.

The research framework was constructed to identify pilots’ SA levels using EEG features after the EEG acquisition experiment in bridge simulator was implemented (Figure 1). Firstly, it was assumed that EEG features and SA were significantly correlated in specific scenes (including poor visibility situation). Consequently, based on SART questionnaire and EEG acquisition technology, the different SA level groups and EEG metrics were separately defined as independent and dependent variables. To ensure the professionalism of questionnaires and measurement accuracy, the SART questionnaires were confirmed through safety engineering and management, maritime supervision, and senior pilot experts to adjust existing measurement items (Jiang et al., 2021).

Secondly, EEG acquisition technology was used to capture pilots’ brain waves, including the θ/β, α/β, θ/(α + θ), (α + θ)/β, and (α + θ)/(α + β) in different brain regions (e.g., F and C), which were used to indirectly infer SA-related constructs (e.g., understanding and prediction). And then the visualization methods (i.e., brain regions activity and time-frequency analysis) are illustrated to preliminarily understand the common EEG patterns and differences between the various SA groups. Although visualization techniques allow analyzing EEG data in an explorative way, a statistical analysis must be performed to determine differences in spatial-frequency analysis as caused by variations in SA levels. Quantitatively, EEG frequency metrics are calculated for pilots in each SA group (high and low) within different regions. The average power-based values of EEG metrics among pilots with high and low SA levels were input into the permutation simulation to compare their associations. After digital and time-domain filtering, the FT-based EEG frequency features were divided into training and testing sets for input to the RF-CNN (Random forest-convolutional neural network) method, which provides the new avenue of classifying the SA for pilots’ screening.

### 2.1. Experiments

#### 2.1.1. Participants

Twenty-five male pilots with normal vision from different pilotages were recruited as experimental participants, who were taking the qualification examination for pilots’ competency in navigation simulators. All pilots were aged from 30 to 45 years old, with an average of 11.3 years pilotage experience. Pilots have provided informed consent about experimental procedures that were approved by the relevant authority.

#### 2.1.2. Situations

**Figure 2:**
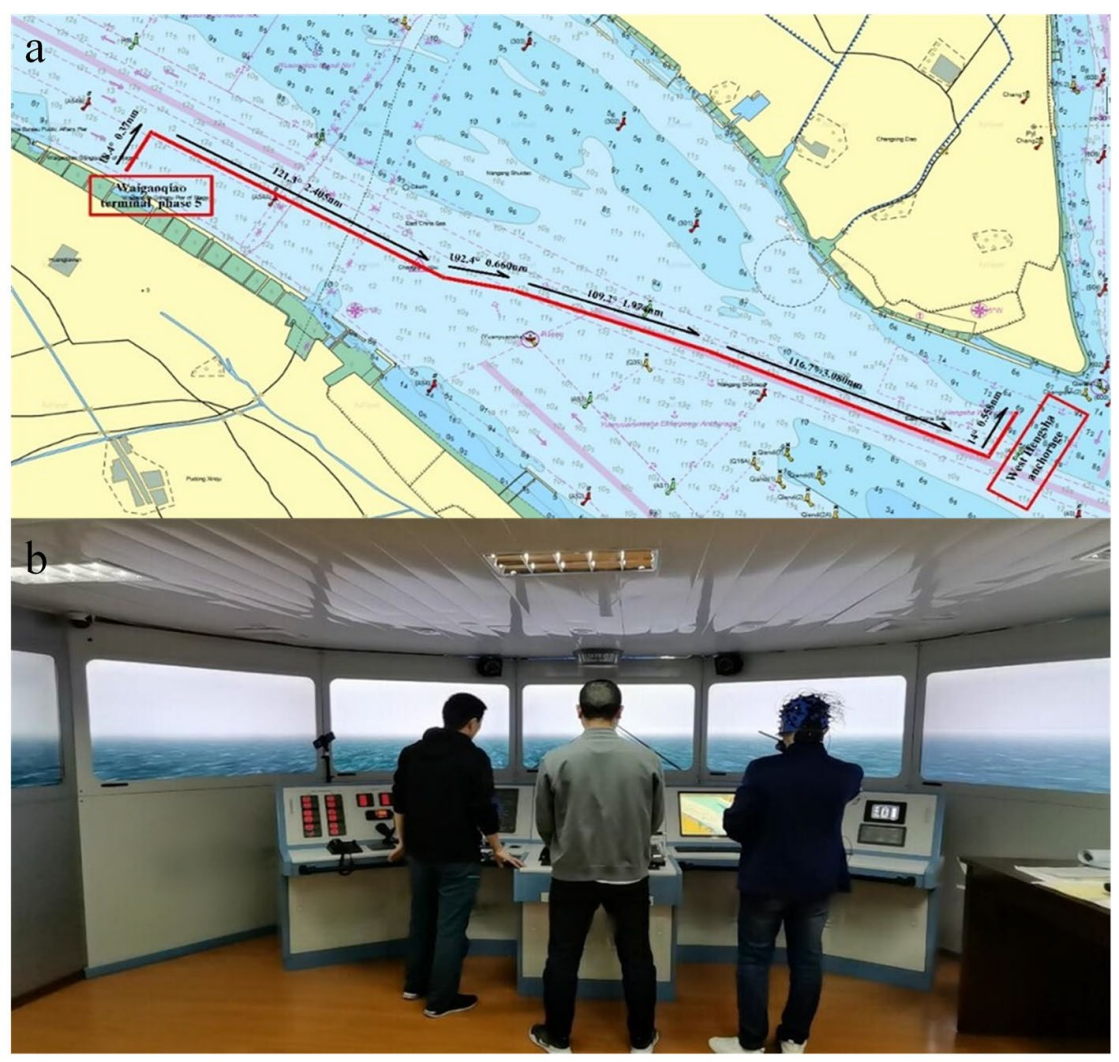
Voyage plan **(A)** and ship pilotage with poor visibility situations in the ship pilotage simulations **(B)**.

The trajectory from Shanghai Waigaoqiao terminal phase 5 to west Hengsha anchorage was selected from the database of qualification examinations, as shown in the Figure 2A. The course and distance of each section of the route from the port to the anchorage are marked according to the industry standard, and the scenes such as ship departure (initial course 18.4 degrees, distance 0.37 nm) and navigation in the fairway (initial course 121.3 degrees, distance 2.40 nm) are included. Poor visibility was included as a mandatory assessment element in the pilotage experiments, as shown in Figure 2B. In addition, the initial conditions included that the type of vessel was uniformly set as 5000TEU container ship, speed of vessel 0 knots, flood tide 1 to 2 knots, and north wind force 3.

#### 2.1.3. Procedure

In this experiment, 25 exams were scheduled for 5 days, each of which tested one participant who acting as a pilot in a three-person exam group. Using the SART questionnaire and EEG acquisition, the experiment was divided into two parts, SA measurement and experiment. The procedure was as follows:

1. In the examination section, the EEG acquisition device (Semi-dry Bitbrain EEG) was calibrated before the experiment. The pilots then used the bridge simulator to sail along a preset navigation route and pass through situations, whereby the pilots conducted at least 40 min of ship pilotage tasks. During this time, a wearable wireless EEG device was used to collect the pilots’ brain waves.
2. The democracy survey was used to pre-judge the pilots’ potential SA level before the experiment. Interviews for the SART questionnaire were implemented to confirm the SA levels in the post-test.

### 2.2. Methods

#### 2.2.1. Data analysis

**Figure 3:**
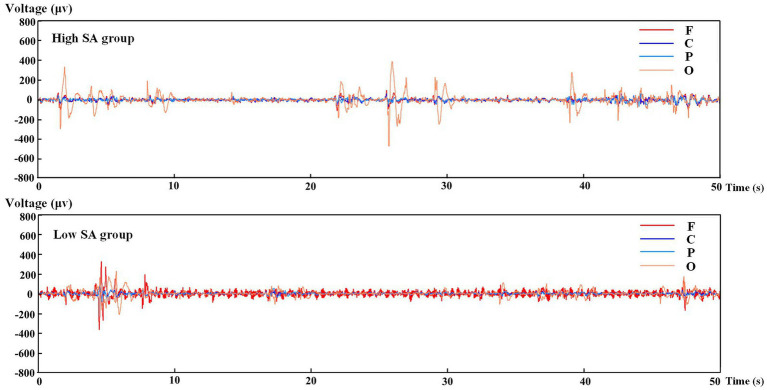
Fragment of the EEG raw data in different SA groups.

The extraction of EEG features involved identifying and calculating the raw data that depend on different SA levels. EEG records neuronal firing in different brain regions by arranging electrodes at corresponding locations in the cerebral cortex, in order to create a dynamic data curve over time. It can be used to reflect the brain activity during a specified time-period and can be classified as spontaneous or evoked EEG signals according to the principle of signal generation (Kaur et al., 2022; Quan et al., 2022). To investigate the cognitive processes of the pilots in a continuous task, rather than the cognitive state at a certain moment, the study of spontaneous rhythmic brain waves was carried out. First, the distribution of the initial EEG data in the temporal and spatial dimensions (including the F, C, P, and O regions) of the pilots in different SA groups was collected and taken for a 50-s period for preliminary analysis, as shown in Figure 3. Where, the voltage value displayed in each region is the average value of the corresponding EEG potential within that region. The distribution of the data revealed that the EEG data of the pilots in the high SA group fluctuated more gently, indicating that they had a better control of the situations during the piloting processes. And the variability of different SA groups in F region was relatively obvious, suggesting that the EEG features in this region may help to distinguish the pilots’ high or low cognitive level. Figure 3 shows the EEG data over a 50-s period, but in practice the device is recording at a sampling frequency of 256 Hz and the total number of samples collected is approximately 1,847,820. In addition, EEG has normal interruptions in capturing brain activity, including other activities from non-neural sources when capturing brain activity, such as ocular artifacts, cardiac artifacts, myoelectric artifacts, and work-frequency interference (Yang et al., 2020), and voltage values above 200 are considered signal artifacts as in Figure 3.

Subsequently, EEG preprocessing is performed by feature extraction to reduce data complexity and remove artifacts. Due to the random, nonlinear, and multi-band characteristics of EEG signals, digital filters are commonly utilized to filter signals from frequency-domain, but they can only filter out the 50 Hz frequency interference in practice. Therefore, indirect processing is required by effective feature extraction methods, including common spatial patterns (CSP), wavelet transform (WT), principal component analysis (PCA), independent component analysis (ICA), autoregressive analysis (AR), and FT. To reduce the data dimensions for effective time-frequency analysis, the FT-based PSD was selected for data processing and feature extraction, and power-based EEG data analysis has been applied in safety critical areas (Wang et al., 2015; Perez-Valero et al., 2021). Specifically, the EEG signals need to be first transformed into the frequency-domain by FT, and then the PSD features are calculated as shown in the following equation:


(1) $$X\left(w\right)=\overset{N=1}{\underset{t=0}{\sum }}x\left(t\right)\exp\left(\frac{-j2\pi wt}{N}\right)\;w=0,1,2,\ldots ,N-1$$

where X(w) is the EEG signal after Fourier variation and *N* is the total number of samples acquisition during the specified time-period.


(2) $$S\left(w\right)=\frac{{\left(\Delta t\right)}^{2}}{N\Delta t}{\left|X\left(w\right)\right|}^{2}\Delta t=\frac{1}{f_{s}}$$

where S(w) is the feature variable of PSD, and Δt and $f_{s}$ denote the time intervals and acquisition frequencies of the samples, respectively.

#### 2.2.2. RF-CNN methods

In the simulation experiments described above, real-time, synchronous EEG data were first collected. The multi-dimension of the EEG signals and its susceptibility to non-cerebral neural activity, which contains a large number of artifacts, make direct analysis of the data difficult (Rabcan et al., 2022). To classify pilots’ SA level using wearable EEG acquisition technology, an RF-CNN method including data input, RF, modified CNN, and verification modules was developed. RF, as an ensemble learning method, carries out voting integration based on the prediction results of decision tree classifiers, and has good accuracy and strong robustness in the identification of noise and outliers (McDonald et al., 2020). CNN algorithm is one of the typical examples of deep learning algorithms, which tend to mathematically represent complex and multi-dimensional classification problems and achieve accurate and fast identification of targets based on good network generalization ability (Zhu et al., 2021; Fan et al., 2022).

Currently, CNN algorithms have been used in direct combination with EEG images, but the identification accuracy is only about 50%. For example, Jie et al. (2014) used CNN alone for image identification of EEG signals and obtained an accuracy of 45%. Based on this, some researchers considered feature extraction of the preprocessed EEG signal and then combined with CNN to carry out the identification of EEG features and achieved better results. For example, Li M. A. et al. (2021) tried to extract the WT features of EEG signals for CNN algorithm training, and the results showed that the final recognition accuracy reached more than 85%. Overall, the use of EEG signal features for RF and CNN model training has achieved good results in safety-related fields and has become an important research trend (Ieracitano et al., 2019; Admiraal et al., 2021). As EEG data involve multiple feature dimensions and are obviously correlated, commonly used machine learning algorithms fail to effectively reduce signal redundancy and relatively lack the ability to identify the internal mechanisms of EEG features. The RF-PCA algorithm is used to rank the importance of the initial EEG data and extract the main components to optimize the features of the input CNN network structure, i.e., to further ensure the overall identification rate of the RF-CNN algorithm by reducing the feature dimensions of the initial EEG data, and to verify the model performance of the optimized method in SA level identification by comparing it with the traditional methods.

##### 2.2.2.1. RF module

As a machine learning method, RFs are effective tools for evaluation and classification (Scornet et al., 2015). The bootstrap sampling technique was used to extract training subsets from the samples that had been preprocessed by digital and time-domain filtering. Decision tree modeling was carried out for each subset (S_1_, S_2_, ⋯, S_k_), and then the classification results were determined by voting according to the principle of majority rule. The objective of the RF was to generate a decision tree dependent on a random variable θ on the basis of data sample X and identification variable Y. Assuming that the identification result of a single decision tree classifier $h\left(x,\theta_{k}\right)$ is $\;h_{i}\left(X\right)$ , the final identification result of the model can be expressed as $\;H\left(X\right)=\frac{1}{k}\overset{k}{\underset{i=1}{\sum }}h_{i}\left(X\right)$ .

The feature importance of the RF was determined by adding noise to a certain feature and considering whether the identification accuracy dropped significantly (Genuer et al., 2010). In the calculation process, the residual mean square of the out-of-bag (OOB) score was used to evaluate the importance of characteristic variables. This can be expressed as:


(3) $$Importance=\frac{\sum \left(errOOB2-errOOB1\right)}{Ntree}$$

where *errOOB1* and *errOOB2* are the OOB identification errors in each decision tree before and after adding noise interference to all sample features, respectively. To determine the optimal parameters, the grid search method was used to ensure model stability. The root mean square error (RMSE) was adopted to prevent the overfitting of feature data. This is calculated as follows:


(4) $$RMSE=\sqrt{\frac{\sum_{i=1}^{n}{\left(y^{obs}-y^{pred}\right)}^{2}}{\sum_{i=1}^{n}{\left(y^{obs}-{\bar{y}}^{pred}\right)}^{2}}}$$

where $y^{obs}$ and ${\bar{y}}^{pred}$ are the observed and predicted values of the corresponding samples, respectively, and *n* is the number of samples. Moreover, because the correlation of initial features might tend to produce partial overlaps of information, PCA was applied to obtain the optimal feature combination:


(5) $$\{\begin{array}{c} F_{1}=e_{11}X_{1}+e_{12}X_{2}+\cdots +e_{1z}X_{z}\\ F_{2}=e_{21}X_{1}+e_{22}X_{2}+\cdots +e_{2z}X_{z}\\ \vdots \\ F_{z}=e_{z1}X_{1}+e_{z2}X_{2}+\cdots +e_{zz}X_{z} \end{array}$$

where $e_{i1}{}^{2}+e_{i2}{}^{2}+\cdots +e_{iz}{}^{2}=1$ *, i = 1,2,*⋯*, z*. No two principal components are related, i.e., $F_{i}\neq F_{j}\;\left(i\neq j\mathrm{;,,,,}i\mathrm{;,,,,}j=1\mathrm{;,,,,}2\mathrm{;,,,,}\cdots \mathrm{;,,,,}z\right)$ . The first principal component F_1_ is determined as being the most different from all linear combinations of the initial importance sequence (X_1_, X_2_, ⋯, X_z_). The second principal component F_2_ is determined as being a linear combination of X_1_-X_z_ that is not related to F_1_ and gives the next-greatest difference from the initial sequence. Similarly, the remaining principal components were sought as new input sets for the CNN.

##### 2.2.2.2. CNN modified module

As a deep learning method, CNNs are often used to deal with binary classification problems with multi-dimension samples (Hersche et al., 2020). Essentially, the CNN algorithm mainly uses convolution, pooling and fully connected networks to alternately extraction, down-dimensioning, and fusion of features from multidimensional EEG data to achieve SA-level identification. The feature fusions for the CNN solved the problem of EEG data filtering and extracting in the identification framework through five structures: input layer, convolutional layer, pooling layer, fully connected layer, and output layer (Li F. H. et al., 2021; Luo et al., 2021). The CNN model with the PCA algorithm was used to transfer the importance rankings and improve convergence efficiency.

###### 2.2.2.2.1. Structure 1

Input layer: The input feature size preset to 13 × 20.

###### 2.2.2.2.2. Structure 2

Convolutional layer: The number of layers depends on the training parameters and convergence speed. In the parameters, the convolution kernel size, i.e., the filtering weights, is usually set to 3 × 3 or 5 × 5 matrices, calculated as follows:


(6) $$g=f\left(\underset{x}{\sum }\underset{y}{\sum }a_{x,y}\times w_{x,y}+b\right)$$

where $w_{x,y}$ denotes the filter weights, *b* is filter bias term, and *f* is activation function.

###### 2.2.2.2.3. Structure 3

Pooling layer: After each convolution process gets the information of different features, layer-by-layer filtering of the convolved features can be achieved based on the pooling processes. The principle of follows closely the forward learning process of the convolutional layer, i.e., the unit matrix corresponding to each feature matrix is computed and normalized based on the movement of the filter from the top-left to the bottom-right corner of the current network (Hu et al., 2019). The pooling layer is used to optimize the parameters of the convolved processes, and the activation functions of the pooling processes, i.e., the nonlinear ReLU, also contributes to reduce the interdependence between the convolution parameters by network sparse matrices, calculated as follows:


(7) $$h^{\left(j\right)}=\max\left(w^{\left(j\right)T}x,0\right)=\{\begin{array}{c} w^{\left(j\right)T}x,w^{\left(j\right)T}x>0\\ 0,w^{\left(j\right)T}x\leq 0 \end{array}$$

where $h^{\left(j\right)}$ is the output of the activation function and $w^{\left(j\right)T}x$ is the multiplier of the weights and corresponding values of the neurons in the j *th* layer. And the maximum pooling process was selected for model training with the following equation:


(8) $$g=\max\left(a_{x,y}\right)$$

###### 2.2.2.2.4. Structure 4

Structure 4. Fully connected layer, where each node is connected to all nodes in the previous layers, and is used to integrate all the features extracted during the convolving and pooling processes. The Softmax is the final output function, which can transform the original output of the neural network into a probability distribution and obtain the recognition probability of the corresponding category. Suppose the original output of the neural network is $\;y_{1},y_{2},y_{3},\ldots \ldots ,y_{n}$ , then the Softmax regression process can be expressed as:


(9) $$Softmax\left(y_{i}\right)=y_{i}^{\prime }=\frac{e^{yj}}{\sum_{i=1}^{n}e^{yj}}$$

To identify the speed of convergence, the cross-entropy validation method is used to describe the distance between the two probability distributions of the output. If the probability distributions of *p* and *q* are given, the formula for their cross-entropy can be expressed as follows:


(10) $$H\left(p,q\right)=-\underset{x}{\sum }p\left(x\right)\log q\left(x\right)$$

For balancing the training time and accuracy of the network, a gradient descent method with error back propagation is also used, i.e., each training is based on a fixed number of samples from the training set only requires parameter updates in opposite direction of the gradient. Assuming J is cost function, the iterative process for each $w_{i,j},b_{i,j}$ is:


(11) $$w_{i,j}^{l+1}=w_{i,j}^{l}-\varepsilon \frac{\partial J}{w_{i,j}^{l}},b_{i,j}^{l+1}=b_{i,j}^{l}-\varepsilon \frac{\partial J}{b_{i,j}^{l}}$$

Where ε is learning rate, $\left(\partial J/w_{i,j}^{l}\right)$ and $\left(\partial J/b_{i,j}^{l}\right)$ are the partial derivatives of errors.

In addition, based on the CNN principle, it is found that each convolution process generates different information, and the convolution layer shows more spatial and frequency features than the fully connected layer. In the feature filtering processes of convolution and pooling, some important features that affect the results are inevitably filtered to ensure the objective requirement of convergence efficiency, which makes the identification performance degraded. Therefore, the PCA method is introduced to improve the CNN structures by reducing the dimensionality of the features after each convolution process, and fusing this reduced data with the pooling process point by point before inputting it to the following convolution layer until the end of training. This CNN modified module not only helps to improve the convergence efficiency, but also integrate important information based on the correlation mechanism to maximize the reflection of EEG time-frequency features in the identification results.

##### 2.2.2.3. Verification module

For the test set containing samples of unknown categories, the confusion matrix of the model output included true positive (TP), false positive (FP), true negative (TN), and false negative (FN) results. To evaluate the identification performance of the RF-CNN method, RF and CNN methods without optimized feature combinations were selected for comparative analysis. The performance evaluation criteria for each classifier were the general accuracy (ACC), true positive rate (TPR), and true negative rate (TNR). These metrics were calculated as follows:


(12) $$ACC=\frac{TP+TN}{TP+TN+FP+FN}\times 100\%$$

(13) $$TPR=\frac{TP}{TP+FN}\times 100\%$$

(14) $$TNR=\frac{TN}{TN+FP}\times 100\%$$

where TP refers to samples with an observed value of 1 and a predicted value of 1, FP refers to samples with an observed value of 0 and a predicted value of 1, TN refers to samples with an observed value of 1 and a predicted value of 0, and FN refers to samples with an observed value of 0 and a predicted value of 0.

## 3. Results

For the purpose of constructing the SA identification model using EEG frequency metrics in different brain regions, groups with different SA levels were first established. To facilitate the analysis, the research hypotheses divide pilots’ SA into two levels based on their SART score: high (above average SART score) and low (below average SART score). According to the SART scores (mean = 20.13, standard deviation = 5.83), the pilots were divided into a high-SA group containing 13 participants (mean = 24.5, standard deviation = 5.13) and a low-SA group containing 12 participants (mean = 15.2, standard deviation = 4.37).

### 3.1. Time-frequency analysis

**Figure 4:**
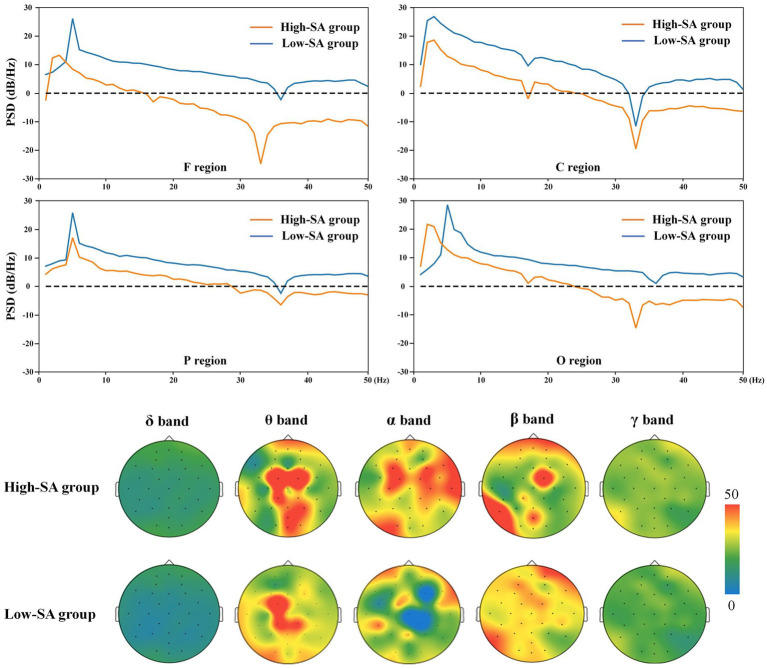
Fragment of the PSDs and brain activity in different SA groups.

To visually compare the EEG pattern of different individuals in the two SA groups, the EEG features are illustrated using time-frequency analysis in the poor visibility situations of the examination. Since the variations in the features of a single channel are most likely the superimposed effects of stimuli in adjacent regions, it is necessary to analyze the time-frequency features combining with spatial-domain (i.e., different brain regions). The spatial-domain represents the dynamic distribution of features in different brain regions over time. Therefore, the PSDs of different brain regions during a time-period was selected, and its interpretation of brain activity by describing the power distribution of different signals in frequency, the results are shown in Figure 4. The PSDs data were higher in the low- than high-SA group, indicating that PSDs may initially reflect brain activity, i.e., brain activity of pilots in the low-SA group is more susceptible to external stimulus. The PSD features in F and C regions had more obvious differences between SA groups, suggesting that identifying different SA levels by features in these two brain regions might have better results.

To further investigate the correlation between SA levels and brain regions activity in specific frequency bands, the average power of δ, θ, α, β, and γ were extracted for activity analysis, and the results are shown in Figure 4. It is shown that the variability of the pilots’ brain activity is mainly concentrated in the θ, α, and β frequency bands, while the δ and γ frequency bands did not vary significantly due to their too-low or too-high frequency ranges, respectively. Moreover, the difference in brain activity between the high- and low-SA groups is more apparent in the F, C, P and O regions, including the C and O regions in the θ band, the C and P regions in the α band, and the F and P regions in the β band. These findings of brain activity analysis provide a preliminary reference and understanding for screening of brain regions in the identification model.

**Figure 5:**
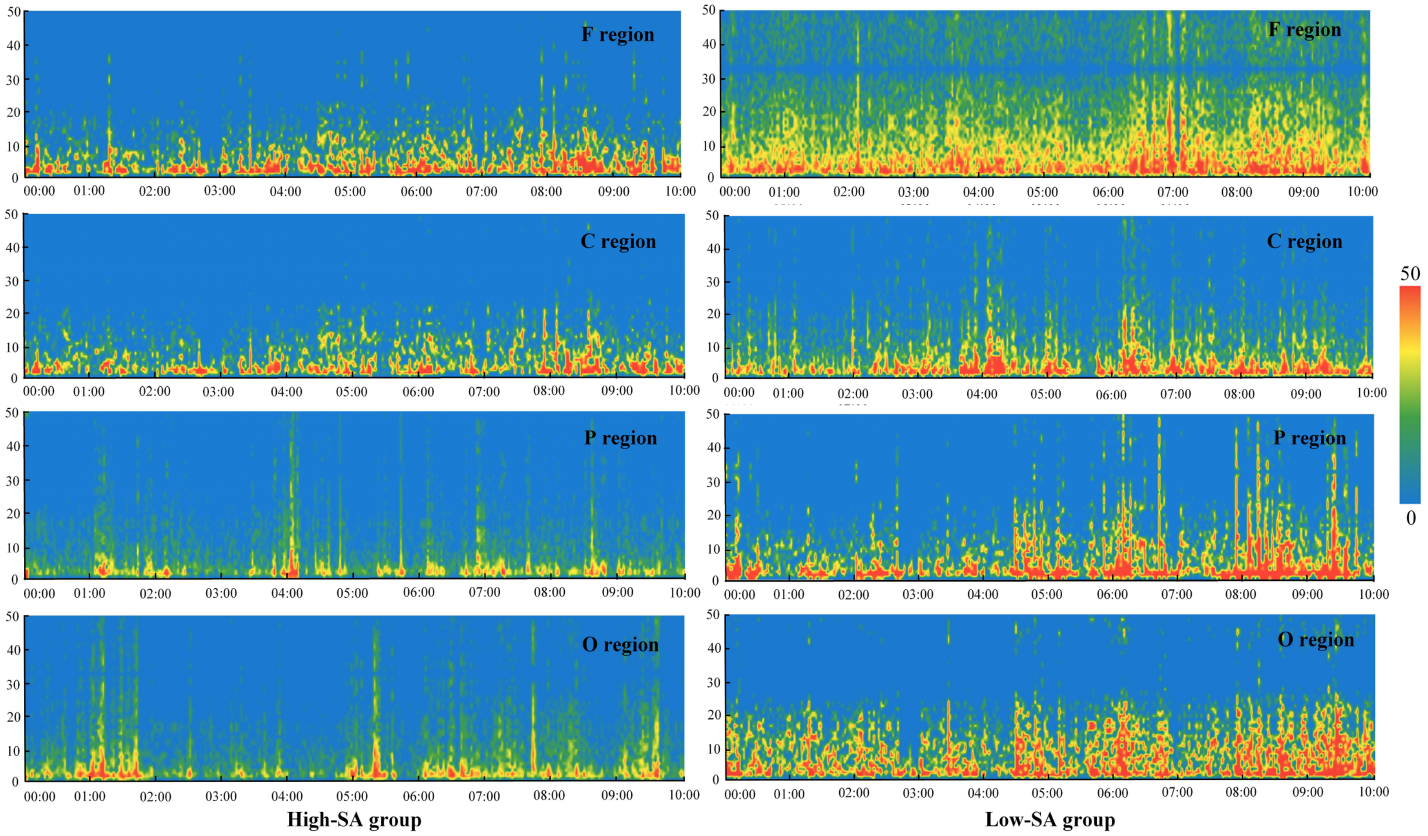
Fragment of the time-frequency in different SA groups.

Time-frequency analysis provides joint information in the frequency- and time-domain to illustrate the time-varying frequency. For understanding the variability of EEG time-frequency features at high- or low-SA levels, a time-period in which poor visibility was selected for feature analysis to obtain the EEG frequency distribution over time in brain regions, as shown in Figure 5. The results show that there are significant differences in the time-frequency features of different SA groups, with the variability in the F and C regions mainly in the high frequency, while the P and O regions are more obvious in the low frequency. As only short time-periods of EEG features were selected for analysis, further statistical tests on the complete task processes are needed. However, there was consistency between the results of EEG time-frequency features and brain area activity, e.g., the finding that the high SA group was relatively inactive in high frequencies in P and O regions in time-frequency was also reflected in brain activity analysis, suggesting that EEG states can be cross-referenced from different feature dimensions. Therefore, the brain region activity and time-frequency analysis in F, C, P and O regions consistently showed relatively significant differences in EEG signals between high- and low-SA groups in θ, α and β frequencies. The visualization outputs and analyses of these recorded EEG features help interpret the results qualitatively to better understand the quantitative SA identification results.

### 3.2. Correlation evaluation

To determine whether these differences are statistically significant, the averages power of the EEG frequency metrics from each SA group are compared across the brain regions using the permutation simulation technique. As before, the θ, α and β frequencies in F, C, P, and O regions are selected for statistical analysis as they are the primary objects with differences in brain activity between high- and low-SA groups in the visualized time-frequency analyses. The statistical differences between the EEG metrics and SA levels in the four regions across the poor visibility situations are calculated in the permutation simulations. Thus, descriptive statistics of five commonly used frequency combination metrics [θ/β, α/β, θ/(α + θ), (α + θ)/β, and (α + θ)/(α + β)] related to cognitive function for the two SA groups and the results of the statistical tests are summarized in Table 1.

**Table 1:** Correlation results in poor visibility situations.

	EEG frequency metrics	High-SA		Low-SA		Permutation results	
		Mean	Std.	Mean	Std.	Welch’s t	*p* value
F region	θ/β	0.376	0.074	0.914	0.164	1.263	0.132
	**α/β**	0.457	0.003	0.752	0.102	1.786	**0.071**^**b**^
	**θ/(α + θ)**	0.253	0.113	0.904	0.036	2.228	**0.048**^**a**^
	**(α + θ)/β**	0.833	0.176	1.666	0.241	2.151	**0.046**^**a**^
	(α + θ)/(α + β)	0.765	0.283	1.254	0.351	2.014	0.350
C region	θ/β	0.395	0.024	0.805	0.245	1.149	0.147
	**α/β**	0.462	0.034	0.812	0.015	2.353	**0.042**^**a**^
	**θ/(α + θ)**	0.257	0.029	0.461	0.054	2.493	**0.026**^**a**^
	**(α + θ)/β**	0.857	0.143	1.617	0.335	2.362	**0.012**^**a**^
	(α + θ)/(α + β)	0.562	0.167	0.865	0.264	0.425	0.618
P region	**θ/β**	0.388	0.146	0.755	0.164	2.339	**0.044**^**a**^
	α/β	0.473	0.217	0.653	0.247	1.324	0.342
	θ/(α + θ)	0.268	0.094	0.509	0.349	1.926	0.435
	**(α + θ)/β**	0.861	0.294	1.408	0.226	2.236	**0.038**^**a**^
	(α + θ)/(α + β)	0.594	0.243	0.816	0.381	1.369	0.236
O region	θ/β	0.373	0.211	0.846	0.429	1.721	0.116
	α/β	0.457	0.214	0.772	0.304	1.355	0.334
	**θ/(α + θ)**	0.264	0.108	0.624	0.241	2.486	**0.027**^**a**^
	(α + θ)/β	0.581	0.143	0.956	0.156	1.583	0.152
	(α + θ)/(α + β)	0.534	0.164	0.721	0.264	1.188	0.435

^a^*p* < 0.1, ^b^*p* < 0.05. The bold values indicate significantly correlated metrics (i.e., *p* < 0.1).

In the statistical analysis of F region, the test results show that α/β (*p* = 0.071 < 0.1), θ/(α + θ) (*p* = 0.048 < 0.05), and (α + θ)/β (*p* = 0.046 < 0.05) are correlated with the SA level. In C region, α/β (*p* = 0.042 < 0.05), θ/(α + θ) (*p* = 0.026 < 0.05), and (α + θ)/β (*p* = 0.012 < 0.05) are significantly correlated with the SA level. The pilot’s SA level also impacts the power of θ/β (*p* = 0.044 < 0.05) and (α + θ)/β (*p* = 0.038 < 0.05) in P region, and θ/(α + θ) (*p* = 0.027 < 0.05) in O region. The poor visibility is not conducive for pilots to obtain any necessary feedforward information related to ship collision hazards in a timely manner and take safe pilotage measures without neglecting stored materials. This demonstrates that they urgently need real-time feedforward information to understand the current situation, which imposes a higher need for the SA level. Due to the average power of the combination metrics of the low-SA group was generally higher than in the high-SA group, the descriptive statistics suggest that the low-SA pilots may be more susceptible to fluctuations in the external navigational environment.

Combined with the correlation evaluation of F, C, P, and O regions in poor visibility situation, these results provide the possibility for follow-up studies to identify pilots’ at-risk SA level using correlated EEG frequency combination metrics. The α/β, θ/(α + θ), and (α + θ)/β in F and C regions may be more conducive to distinguish this ability between pilots with different SA levels. Moreover, to reduce the data volume and noise, the mean and median power of EEG frequency metrics within a sliding time window of 5 s (i.e., epoch length) was used for data separation and feature extraction. The calculated results were then considered as the features to be selected in the subsequent identification model, as listed in Table 2.

**Table 2:** List of calculated features.

Signal type	Features
α/β	Average power of F region in 5 s (FM1)
	Median power of F region in 5 s (FP1)
	Average power of C region in 5 s (CM1)
	Median power of C region in 5 s (CP1)
θ/(α + θ)	Average power of F region in 5 s (FM2)
	Median power of F region in 5 s (FP2)
	Average power of C region in 5 s (CM2)
	Median power of C region in 5 s (CP2)
(α + θ)/β	Average power of F region in 5 s (FM3)
	Median power of F region in 5 s (FP3)
	Average power of C region in 5 s (CM3)
	Median power of C region in 5 s (CP3)

### 3.3. SA identification

**Figure 6:**
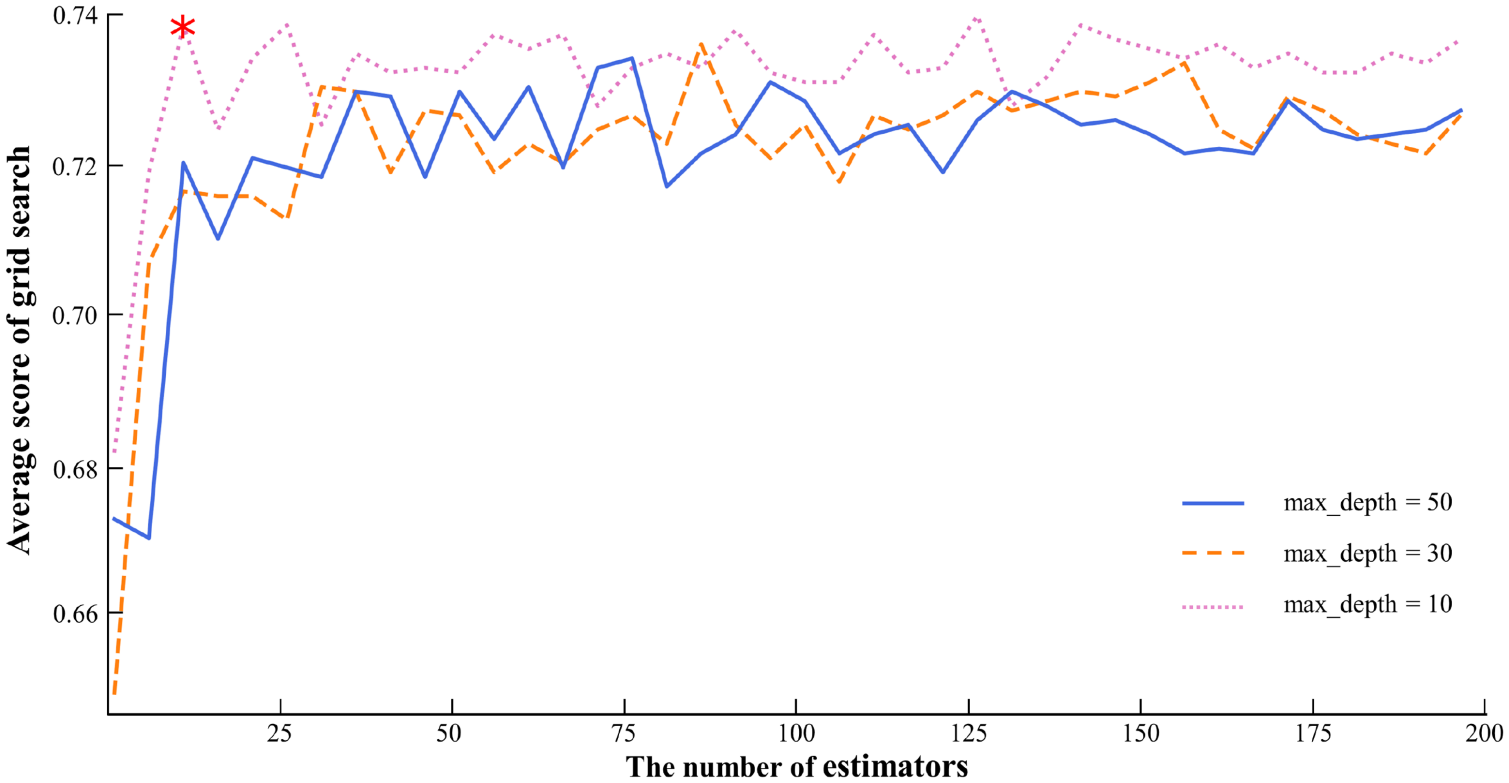
Parameter optimization of the RF method.

In this study, a nonlinear RF-CNN method was used for binary identification of the cognitive state, i.e., high- and low-SA levels, based on the frequency features extracted from the EEG data of the ship pilotage experiment. First, after preprocessing the data with time-frequency analysis, the EEG features associated with pilot SA levels were classified into 12 categories based on the results of the correlation evaluation, as listed in Table 2. The features were then separated into training and testing sets before the RF-CNN method was constructed. The training set was randomly selected, accounting for 75% of the feature samples. Figure 6 shows the optimal parameters of the RF method obtained using the grid search method. The maximum search efficiency of 0.7399 was obtained with 11 estimators and a maximum depth of 10. Subsequently, the optimal parameters of RF method were confirmed, and the initial feature importance ranking based on the average score was obtained (see Table 3).

**Table 3:** Initial feature importance score and ranking.

Number	Feature	Score	Number	Feature	Score	Number	Feature	Score
1	FM2	0.225	5	CM1	0.082	9	CM3	0.063
2	CP1	0.094	6	FP3	0.079	10	FM1	0.059
3	CM2	0.086	7	CP2	0.070	11	CP1	0.051
4	FP2	0.085	8	FM3	0.064	12	CP3	0.048

To prevent the overfitting of the identification model, the feature with the lowest importance score in Table 3 was eliminated, and then the remaining feature data were input back into the RF method. After 11 iterations of this feature screening process, the RMSE and relative error (RE) were obtained for 1–11 features, as listed in Table 4. The results show that the RMSE and RE of the identification were minimized with 10 features. Therefore, CP1 and CP3 were eliminated, and 10 valid features were retained. Moreover, because of the possibility of overlapping information in the EEG frequency combination features of the same individual, the PCA algorithm was used to reduce the dimension of the features. The first 10 features in the importance ranking were calculated by the PCA algorithm, where $X_{1}$ - $X_{10}$ corresponded to the features with importance ranking 1–10. The first six principal components were selected as extraction criterion, as these gave cumulative contribution to the rate of variance of more than 87.7%. The eigenvalues of the correlation coefficient matrix and the contribution rate of the principal components are listed in Table 5. These six principal components are:


$$\begin{array}{l} F_{1}=0.1226X_{1}+0.0706X_{2}+0.2358X_{3}+0.1079X_{4}+0.0992X_{5}+\\ 0.0693X_{6}+0.0716X_{7}+0.1063X_{8}+0.0650X_{9}+0.0625X_{10} \end{array}$$

$$\begin{array}{l} F_{2}=0.2015X_{1}+0.0979X_{2}+0.1352X_{3}+0.1052X_{4}+0.1147X_{5}+\\ 0.0713X_{6}+0.0676X_{7}+0.1012X_{8}+0.0450X_{9}+0.0705X_{10} \end{array}$$

$$\begin{array}{l} F_{3}=0.1526X_{1}+0.1203X_{2}+0.0718X_{3}+0.1653X_{4}+0.1201X_{5}+\\ 0.0732X_{6}+0.0685X_{7}+0.1041X_{8}+0.0732X_{9}+0.0523X_{10} \end{array}$$

$$\begin{array}{l} F_{4}=0.1206X_{1}+0.2013X_{2}+0.1017X_{3}+0.1318X_{4}+0.0797X_{5}+\\ 0.0552X_{6}+0.0765X_{7}+0.0671X_{8}+0.1092X_{9}+0.0643X_{10} \end{array}$$

$$\begin{array}{l} F_{5}=0.2156X_{1}+0.1501X_{2}+0.0827X_{3}+0.1018X_{4}+0.0787X_{5}+\\ 0.0912X_{6}+0.0615X_{7}+0.0671X_{8}+0.1022X_{9}+0.0563X_{10} \end{array}$$

$$\begin{array}{l} F_{6}=0.1906X_{1}+0.1315X_{2}+0.1037X_{3}+0.0918X_{4}+0.1253X_{5}+\\ 0.0652X_{6}+0.0890X_{7}+0.0761X_{8}+0.1091X_{9}+0.0573X_{10} \end{array}$$

$$\begin{array}{l} F_{i}=0.2862F_{1}+0.1965F_{2}+0.1616F_{3}+\\ 0.1317F_{4}+0.1142F_{5}+0.1097F_{6} \end{array}$$

**Table 4:** Error values with various numbers of features.

Number of features	RMSE	RE	Number of features	RMSE	RE
1	0.875	0.498	7	0.751	0.359
2	0.802	0.427	8	0.704	0.312
3	0.765	0.396	9	0.675	0.297
4	0.735	0.371	10	0.627	0.209
5	0.732	0.363	11	0.702	0.282
6	0.741	0.370			

**Table 5:** Principal components of features.

Component	Eigenvalues	Contribution (%)	Cumulative contribution (%)
F_1_	5.193	25.108	25.108
F_2_	4.109	17.233	42.341
F_3_	1.825	14.176	56.517
F_4_	1.372	11.548	68.065
F_5_	3.014	10.016	78.081
F_6_	2.917	9.624	87.705

**Figure 7:**
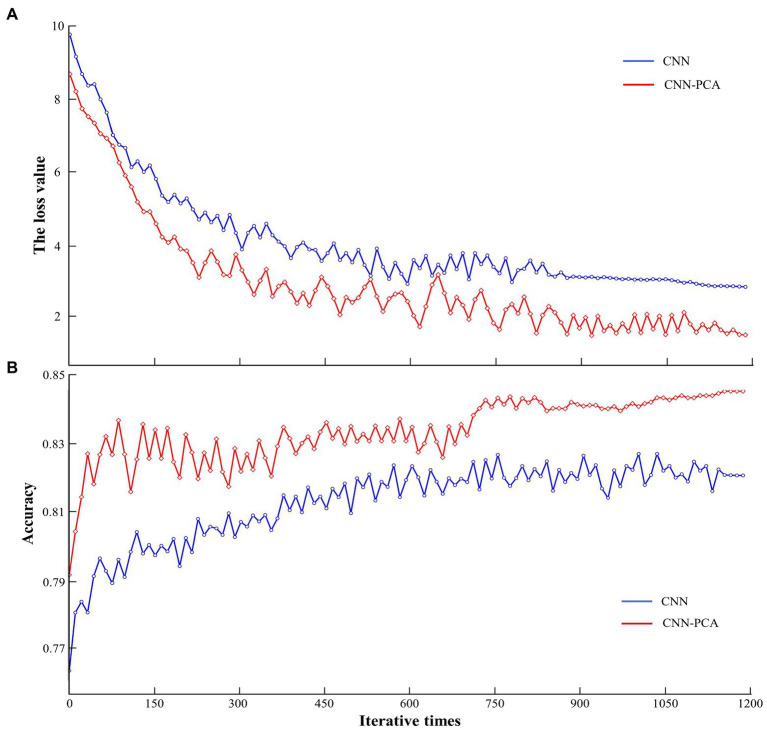
The loss value **(A)** and accuracy **(B)** of CNN modified method training.

The first six principal components were extracted in the form of feature combinations and used to construct new input sets for the CNN modified model. Before the model training, the initial hyperparameter values were preset, including the batch size set to 20, the convolutional kernel set to 5 × 5, the maximum pooling to 3 × 3, the network learning rate to 0.1 and the weight decay parameter to 0.0001. The results indicate that the learning rate is reduced by 50% when the initial increment of the loss function is greater than 25%. Figure 7A shows the trained loss values reach the expected level when the iterations exceed 900, and the convergence rate and loss values of the CNN model modified by PCA are better than those of the traditional CNN method. Further, the identification accuracy of the two methods is compared, and Figure 7B shows that the identification rate of CNN modified method remains around 84.8% after more than 1,200 iterations. Thus, in the poor visibility, the PCA method contributes to improve the convergence rate and identification accuracy of the traditional CNN method is verified.

**Figure 8:**
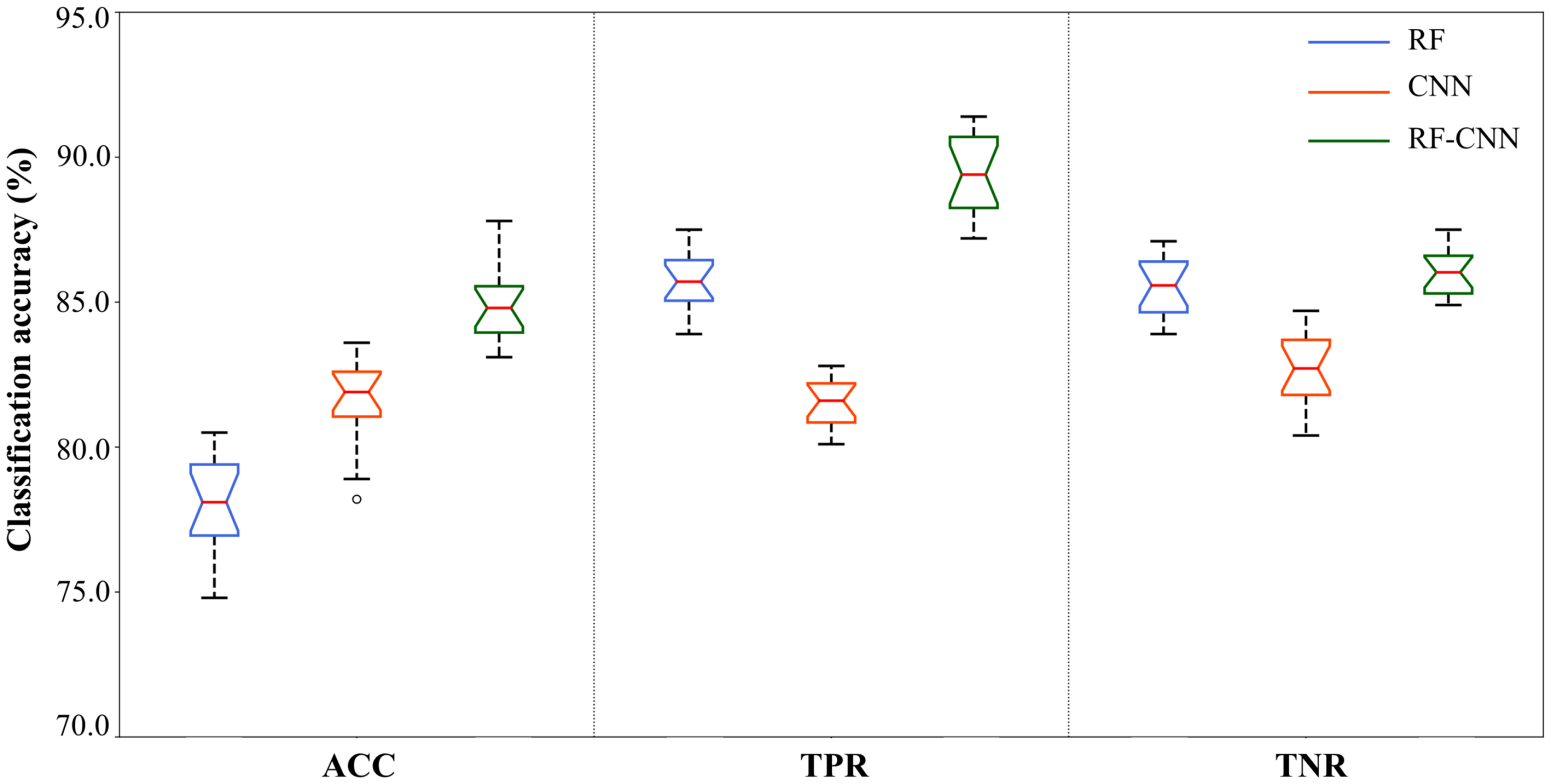
Performance evaluation of the three methods.

In the comparative analysis of the RF, CNN, and RF-CNN methods, three performance evaluation metrics were used to evaluate the effectiveness of the optimized feature combinations. Figure 8 presents the distribution of the classification accuracy measure for the three classifiers using different performance metrics. In each box, the bottom and top edges indicate the 25th and 75th percentiles, respectively, and the center mark denotes the median. Using the optimal EEG frequency feature combinations in the RF-CNN methods, the average ACC over 1,200 calculations reached 0.848, while the TPR was 0.894 and the TNR was 0.860. Moreover, to facilitate the evaluation of the classification performance of the method, the Matthews correlation coefficient (MCC), F1-score and Kappa indexes were selected for analysis and found to be within reasonable values. In general, the RF-CNN outperformed the RF and CNN methods without feature optimization.

**Figure 9:**
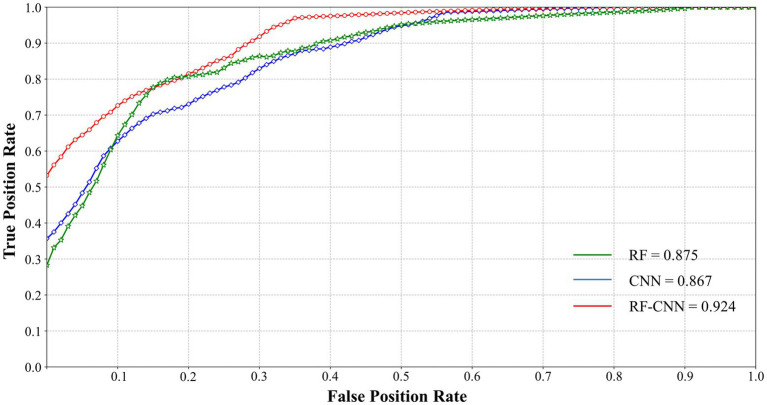
ROC curve of the three methods.

Because the receiver operating characteristic (ROC) curves can intuitively observe the accuracy of the classification method through the graph, and usually combined with under the curve (AUC) to solve the evaluation problem of binary classification (Omar and Ivrissimtzis, 2019). With the optimal RF-CNN parameters, we retrained and retested ROC and the area AUC of the RF-CNN algorithms for comparative analysis. The results are shown in Figure 9. The AUC scores were 0.875, 0.867, and 0.924 for the RF, CNN, and RF-CNN algorithms, respectively, demonstrating the better stability of the RF-CNN. The superior sensitivity and specificity of the RF-CNN algorithms are verified by the TPR and TNR scores, respectively. Table 6 reports the performance of the three classification algorithms using the evaluation methodology described above. Using the optimized features as input data, the RF-CNN algorithm achieved an average accuracy of 0.848, average sensitivity of 0.894, average specificity of 0.860, and an AUC score of 0.924. These results demonstrate that the RF-CNN with optimized parameters achieves better performance than traditional algorithms in terms of the identification of EEG frequency combination features with different SA levels. This provides an important cognitive avenue for the construction of a screening and evaluation model for pilots’ competency.

**Table 6:** Comparative analysis of performance metrics.

Classification algorithm	Accuracy	Sensitivity	Specificity	AUC
RF	0.781	0.857	0.855	0.875
CNN	0.816	0.815	0.827	0.867
RF-CNN	0.848	0.894	0.860	0.924

## 4. Discussion

The results highlight that the RF-CNN method achieves good SA identification using six principal components of EEG features (Table 5). The EEG data of 25 pilots were collected during the whole experimental process based on pilots’ competency in poor visibility situations. Moreover, the poor visibility was designed to appear randomly in normal navigation during the simulation experiment. With respect to the fundamental analysis of EEG data, a permutation simulation was used to quantify the significance of the correlation between EEG cognitive state, as visualized in the form of brain activity and time-frequency analysis, and SA levels, as measured by SART. As the window width of short-time Fourier transform (STFT) is fixed and cannot be adaptively adjusted, it is usually applicable to the analysis of stable signals. WT is then proposed to observe the partial features of the signal through time windows of adaptive widths varying with frequency, but is often considered as a single power signal in the spatial dimension. Therefore, combining the time-frequency and spatial features of EEG signals, the frequency combination features of different brain regions in the time dimension are extracted for SA identification. The EEG frequency features were then extracted from the associated metrics and divided into overlapping 5 s epochs after digital and time-domain filtering. To prevent possible data overfitting and information overlap, the initial number of input features was determined to be 10 based on the minimum RMSE and RE values of the RF model, and the PCA algorithm was used for further EEG feature combination to obtain six major components as the final input set of the CNN model. Moreover, PCA was applied to optimize the network structure of the CNN model to improve the training convergence efficiency and accuracy, and the feasibility of the above methods has been verified by comparing the results with traditional methods (see 3.3 for details). The RF-CNN method was then used to obtain the identification accuracy of the at-risk cognitive competency (i.e., low SA level). Comparative analysis of the three-performance metrics shows that the proposed method outperforms two other traditional methods that do not use feature optimization.

The results of this study confirm that the at-risk cognitive state can be effectively detected with high accuracy, sensitivity, and specificity using EEG frequency features combined with an appropriate identification framework. The identification results obtained by the RF-CNN, RF, and CNN algorithms with different types of features are shown in Figure 8. We can observe that a combination of the top-nine most important features facilitates an improvement in classification accuracy, from 78.1% using the RF without feature optimization to 84.8% using RF-CNN. Comparing the RF and CNN algorithms, which used the same feature data as their inputs, the latter produced better ACC, TPR, and TNR values, which verifies the applicability of the CNN for nonlinear multidimensional sample sets of EEG data in poor visibility situations. Therefore, we can conclude that an effective RF-CNN method for identifying at-risk cognitive state should include a strong classifier after PCA optimization (i.e., CNN modified module) and input data that have been refined by the combination of salient EEG frequency features in different brain regions (i.e., RF module).

An accurate comparison with the results from other studies in which EEG was used is difficult, because each study used a different method of simulation, a different set of EEG features, and a different classification of SA groups. The main limitation of the current study involves the performance issue of EEG acquisition devices. When the pilot is in a simulation experiment, the EEG devices are susceptible to environmental factors and physiological artifacts that generate large amounts of clutter. Although digital and time-domain filtering helps with feature extraction, it inevitably affects the significance level of the correlation results. Moreover, regarding feature extraction, the identification accuracy is associated with the correlation metrics and epoch lengths of EEG data in poor visibility situations. This study shows that pilots with low SA levels are susceptible to environmental risk factors resulting in significant EEG fluctuations, as expressed by a total of 14 EEG frequency combination features in F and C regions with an epoch length of 5 s. However, the SA level is not only correlated with EEG time-frequency features but is also related to other physiological measurement metrics, as confirmed in previous studies (Mehta et al., 2018; Zahabi et al., 2021). Therefore, the identification processes of at-risk cognitive states are complicated, and require further investigation considering multiple fusion metrics such as heart rate variability (HRV), electrocardiograph (ECG) and eye-tracking signals with different epoch lengths, as well as multiple classifications of SA groups (Paulus and Remijn, 2021; Xiong et al., 2021; Ren et al., 2022).

## 5. Conclusion

The results using the proposed RF-CNN method confirm that it is feasible to identify pilots’ at-risk cognitive competency (i.e., low SA levels) using EEG features. Specifically, the EEG data of 25 ship pilots were obtained from bridge simulation experiments in poor visibility situations for the training of the identification model, containing RF, modified CNN and validation modules. Six EEG principal components were produced by the PCA after RMSE and RF correction to obtain the optimal model training sets, with the cumulative contribution rate of more than 87.7%. The experimental results demonstrate that the proposed feature combinations enhance the classification performance over that of RF and CNN, which do not employ features optimization, demonstrating the potential for our approach to be used in the computer-aided screening of pilots’ cognitive competency. Considering the universality of the proposed method, future research will focus on the verification and application of the identification model in different emergency situations (e.g., ship departure, two-ship crossing, and anchoring) with multiple fusion metrics (e.g., HRV, eye-tracking). Therefore, this study yields not only immediate benefits in monitoring cognitive competency and preventing unsafe behaviors, but also long-term benefits in opening new avenues for the construction of evaluation systems for the physical and mental competency of ship pilots.

## Data availability statement

The raw data supporting the conclusions of this article will be made available by the authors, without undue reservation.

## Ethics statement

Ethical review and approval was not required for the study on human participants in accordance with the local legislation and institutional requirements. The patients/participants provided their written informed consent to participate in this study.

## Author contributions

All authors listed have made a substantial, direct, and intellectual contribution to the work and approved it for publication.

## Funding

This work was supported by the National Natural Science Foundation of China (Grant No. 71503166) and the Jinhua municipal public welfare technology application research project (Grant No. 2022-4-014).

## Conflict of interest

The authors declare that the research was conducted in the absence of any commercial or financial relationships that could be construed as a potential conflict of interest.

## Publisher’s note

All claims expressed in this article are solely those of the authors and do not necessarily represent those of their affiliated organizations, or those of the publisher, the editors and the reviewers. Any product that may be evaluated in this article, or claim that may be made by its manufacturer, is not guaranteed or endorsed by the publisher.
